# Mental health and psychosocial support for the war-wounded: A retrospective cohort study from the Democratic Republic of Congo, Mali and Nigeria

**DOI:** 10.1371/journal.pone.0268737

**Published:** 2022-05-24

**Authors:** Ida Andersen, Rodolfo Rossi, Polycarp Kyaave Nyamkume, Ives Hubloue

**Affiliations:** 1 Health Unit, International Committee of the Red Cross, Geneva, Switzerland; 2 Research Group on Emergency and Disaster Medicine, Vrije Universiteit Brussel, Brussels, Belgium; Yamaguchi University: Yamaguchi Daigaku, JAPAN

## Abstract

**Background:**

For more than 150 years, war surgery has been at the heart of the humanitarian assistance offered by the International Committee of the Red Cross (ICRC) in conflict zones around the world. Mental health and psychosocial support (MHPSS) is increasingly recognized as an integral part of the medical care offered to this highly vulnerable group of patients. This study seeks to identify patient characteristics associated with high distress prior to MHPSS and predictors of improvement following it.

**Methods:**

Between October 2018 and April 2020, 2,008 weapon-wounded patients received MHPSS in ICRC-supported hospitals in the Democratic Republic of Congo (DRC), Mali and Nigeria. The 21-item Depression and Anxiety Scale (DASS21), the Impact of Events Scale Revised (IES-R) and the ICRC functionality scale for Africa were administered before and after the MHPSS response. Logistic regression models were used to measure associations between outcome and exposure variables. Data was initially collected for monitoring purposes and analyzed retrospectively for the sake of this study.

**Results:**

The main reasons for surgery were firearms (65%), other weapons (13%) and mines (5%). Linear trends were found between increasing number of days between violence and first consultation and decreased likelihood of presenting high levels of anxiety (aOR 0.75, *p* = 0.014), and stress (aOR 0.78, *p* = 0.032). Violence committed by military/armed group was associated with increased likelihood of reporting high levels of anxiety (aOR 2.47, *p* = 0.047). On the IES-R, high scores at baseline were more likely to be found among illiterate patients (aOR 0.08, *p* = 0.042) and having been wounded by firearms considerably increased the likelihood of reporting high levels of PTSD (aOR 21.34, *p* = 0.035). Following MHPSS, 92.28% of the patients showed a reduction in symptoms on the DASS21, 93.00% showed a reduction in symptoms on the IES-R and 83.04% showed an improvement on the ICRC Africa functioning scale. On the DASS21, factors negatively associated with improved anxiety included lack of social support (aOR 0.17, p = 0.047) and suffering from a chronic medical/physical condition (aOR 0.40, *p* = 0.013). Patients with reduced IES-R scores were more likely to have a high level of education (aOR 8.95, *p* = 0.029) and to have received MHPSS that lasted between 22 and 30 days (aOR 8.73, p = 0.008). Predictors of improved functioning included being 35–44 years of age (aOR 3.74, p = 0.004) and suffering from a severe or chronic medical condition (aOR 1.66, p = 0.044).

**Conclusions:**

Clinical implications of this study include the increased involvement of family and other caregivers in the MHPSS and longer-term follow-up of patients with severe and/or chronic medical conditions. Further research is needed with regard to joint psychological and physical outcomes, the role of the patient’s education level and the personal styles and techniques used by the counsellors.

## Background

For more than 150 years, war surgery has been at the heart of the humanitarian assistance offered by the International Committee of the Red Cross (ICRC) in conflict zones around the world [1]. Protection and assistance to weapon-wounded constitute the first of the four Geneva Conventions [2]. Often life-saving, this assistance pertains to weapon bearers and civilians alike. In recent years, there has been an increased acknowldgement of the psychological needs of weapon-wounded patients stemming from reactions associated with the physical trauma, reactions to medical procedures, reactions to changes in one’s physical condition and reactions and readjustments to one’s social and family environment [3].

A limited number of studies exist on this topic. An analysis of 953 surgical patients in a hospital in Afghanistan described injury patterns but did not consider psychological factors related to recovery [4]. A study of 138 recently amputated patients in Brazil found an association between psychological problems, lack of independence and associated chronic diseases [5]. Also, a recent randomized controlled study of 756 day-surgery patients in Sweden documented that low preoperative mental health was associated with poorer postoperative recovery [6]. Finally, a case study from the United States pointed out the need to combine medical and psychological care to address pain and PTSD among gunshot survivors [7] and highlighted the important role of catastrophizing thoughts in the patient’s experience of pain. These findings point to the interdependency between physical and mental health, particularly when it comes to recovering from war surgery.

To further address the gap in research and informed clinical practice with regard to mental health and psychosocial support (MHPSS) for war-wounded patients, this study seeks to identify patient characteristics associated with high distress prior to MHPSS and predictors of improvement following it.

## Methods

### Study design

This is a retrospective cohort study of 2,008 war-wounded patients who received MHPSS in ICRC-supported hospitals in the DRC, Mali and Nigeria between October 2018 and April 2020. Data was initially collected for monitoring purposes and analyzed retrospectively for the sake of this study.

### Study sites and participant selection

Between October 2018 and April 2020, the ICRC supported surgical care to war-wounded patients in 44 hospitals in conflict settings across Africa. In ten of these hospitals, MHPSS was an integrated component. However, systematic monitoring was only carried out in nine of these hospitals–four in the DRC, four in Mali and one in Nigeria. In these nine facilities, 3,386 patients received surgical care following war wounds and an additional 5,968 patients underwent surgery for other reasons. All war-wounded patients were approached by a counsellor at some point during the hospitalization and offered MHPSS. As many as 2,008 war-wounded patients wished to receive MHPSS during this period and were all included in the study.

### Sources of data and variables

All data used in this study derives from an ICRC MHPSS Excel database containing routinely collected information regarding the counsellor (one variable), patient demographics (ten variables), trauma history (nine variables), type of support received (five variables) as well as pre- and post-assessment scores of psychological distress and daily functioning. To assess psychological distress, the Depression, Anxiety and Stress Scale with 21 items (DASS21) was used in the DRC and Mali, while the Impact of Events Scale Revised (IES-R) was used in Nigeria. To assess daily functioning, the ICRC functionality scale for Africa was used in the DRC, Mali and Nigeria. For a detailed description of the scales used in ICRC MHPSS programmes, see Andersen *et al*., 2020 [8].

Data was initially recorded on paper files by the counsellors within the hospital and subsequently entered into the ICRC MHPSS Excel database by ICRC MHPSS data clerks in the nearest ICRC sub-delegation.

### The MHPSS intervention

ICRC MHPSS programmes for weapon-wounded patients in Africa are carried out in collaboration with the Ministry of Health and/or the National Red Cross Society in the given country. An ICRC MHPSS team consisting of international and national psychologists trains and supervises counsellors working inside the surgical wards. The counsellors offer awareness-raising sessions for health staff, patients and accompanying caregivers on various topics related to MHPSS needs and services available within the hospital. The main activity of the counsellors is to provide individual counselling that is carried out in three phases–pre-assessment, follow-up sessions and post-assessment.

#### Pre-assessment

During the first phase, a counsellor approaches the newly arrived war-wounded patient to establish first contact, answer any immediate questions and offer reassurance and emotional support, as needed. A few days later, when the physical injury is stabilized and the patient is more calm, the counsellor approaches the patient again for an in-depth psychological assessment. Information about the patient and the trauma history is collected along with psychometric tools measuring levels of distress and functioning. For patients who report psychological symptoms or reactions during the assessment and express interest in receiving further support, a psychological treatment plan is developed focusing on the main clusters of symptoms.

#### Individual counselling sessions

The second phase of the service delivery is the follow-up sessions during which the treatment plan is carried out. Together with the patient, the counsellor addresses the most pressing difficulties that most often fall within four main domains:

1. First, reactions associated with the physical injury and violent circumstances surrounding it may include trauma-related symptoms such as shock, anxiety and anger. At this acute stage, the counsellor can help the patient and accompanying caregivers by answering immediate questions about the unfamiliar hospital environment and providing psychoeducation to normalize reactions to violent events, as well as calming techniques such as breathing exercises.
2. Second, reactions to medical procedures are common as patients may experience intense fear of pain or death to the point of refusing surgery or other medical procedures. In these situations, the counsellor may be present while the medical team explains in detail the necessity of the medical treatment and the different stages of recovery. The counsellor may then stay with the patient and caregivers afterwards to repeat some of the information given by the medical staff and offer reassurance.
3. Third, reactions to changes in one’s physical condition are common given the life-changing impact that the injury and potential amputation has on the patient. Despair and hopelessness may arise as the patient struggles to project him or herself into a meaningful future. The counsellor may invite the patient to explore coping mechanisms used in the past and adopt a problem-solving approach to overcome obstacles for utilizing these coping mechanisms, i.e. socializing in the present.
  In some instances, catastrophizing thoughts and fear of re-experiencing intense pain may lead the patient to restrict movement despite medical advice to perform certain rehabilitative exercises. The counsellor may then collaborate with the medical team to understand and help the patient manage his or her thoughts, feelings and behaviour on the path to recovery.
  Abuse of pain-relieving drugs is also a concern that the counsellor helps the medical team address through information about these risks. Additionally, the counsellor supports the patient in managing pain through self-distraction, relaxation techniques, praying or other ways of coping.
4. Fourth, reactions and readjustments to one’s social and family environment are likely to appear as the patient anticipates the challenges of a prolonged recovery and/or long-lasting disability. The counsellor may help to focus on what the patient has some control over and channel concerns into optimizing adherence to treatment, from following dietary and hygiene plans to medication intake and wound-cleaning.

Change in social status and family role, including fear of abandonment, are themes that the counsellor can discuss with the patient individually or through family sessions. Some counsellors collaborate with former patients who participate in awareness-raising activities or individual sessions with current patients to talk about how they managed to overcome their physical and psychological wounds.

#### Post-assessment

Phase three is the post-assessment and closure, which most often take place when the patient is about to be discharged. During this session, the counsellor makes a summary of the topics discussed in all the sessions and administers the psychometric tools used during the pre-assessment phase, pointing out the main areas in which the patient has reduced his or her distress and/or improved his or her daily functioning. The counsellor then addresses any last questions or concerns, informs the patient about MHPSS and other services available outside of the hospital and proceeds to close the patient file. When pertinent, the medical team refers the patient to physical rehabilitation services in ICRC-supported physical rehabilitation centres.

### Data management and statistical analysis

All categorical data were numerically coded. Quantitative/continuous variables (i.e. pre- and post- scores) were either kept as such or categorized depending on the type of analysis. Categorization of continuous variables was done either by identifying the median to divide the study participants into two even-sized groups or by using established clinical cut-offs.

The dataset was created in Microsoft Excel with two independent data clerks to check against potential typing mistakes. The electronic dataset was protected by a password, which was changed every three months. The dataset was transferred to STATA™, version MP 16.0, for analysis.

All quantitative variables were explored by defining their means (and standard deviation), medians and quartiles. Comparisons of means were tested through the t-test, and the corresponding p-value was reported; 95% confidence intervals (95% CI) were calculated around means and means differences. Categorical variables were explored through percentages and tested using the Chi^2^ test to retrieve the corresponding p-value; 95% CIs were calculated around these percentages.

To measure associations between variables (crude and multivariable), logistic regression models were fitted to calculate odds ratios (OR) with corresponding 95% CIs and p-values from the Wald test. All variables were initially explored in a crude analysis before being inserted in a multivariable model. Results from logistic regression were only presented if they were found statistically or clinically significant.

## Results

### Characteristics of the patients

The 2,008 war-wounded patients included in the study (Table 1) resided in the DRC (39%), Nigeria (35%) and Mali (27%). The majority were male (78%) and registered as civilians (90%) as opposed to weapon bearers. Their age varied from 0–17 (12%), 18–24 (23%), 25–34 (29%), 35–44 (20%) to 45–85 (16%), while education level varied from illiterate (45%), primary (32%), secondary (19%) to university level (4%). The main reasons for surgery were firearms (65%), other weapons (13%) and mines (5%). Most of the violence had taken place while going somewhere (40%), in the patient’s home (31%) or at the patient’s workplace/school (17%). The main perpetrators were military/armed groups (67%) and unknown civilians (13%). Other factors of vulnerability highlighted by the patients during the first session included having suffered destruction or loss of property and/or income as a result of violence (35%), suffering from a severe or chronic medical condition (28%) and having experienced the natural death of a loved one more than two years ago (16%) or less than two years ago (9%). The timing of the MHPSS activity varied from 0–2 days since exposure to violence (21%), 3–14 days (55%), 15–90 days (18%), 91–365 days (3%) to >365 days (4%).

**Table 1:** Characteristics of the study population and the MHPSS.

	N	%
**Country (N = 2,008)**		
Democratic Republic of the Congo	791	39.39
Mali	538	26.79
Nigeria	679	33.81
**Gender (N = 2,008)**		
Male	1,571	78.24
Female	437	21.76
**Age (N = 2,001)**		
0–17	241	12.04
18–24	467	23.34
25–34	579	28.94
35–44	394	19.69
45–85	320	15.99
**Education level (N = 1,993)**		
Illiterate	903	45.31
Basic	640	32.11
Medium	371	18.62
High	79	3.96
**Weapon bearer (N = 2,008)**		
No or not disclosed	1,831	91.19
Yes	177	9.81
**Civil status (N = 1,460)**		
Single (incl. children)	542	37.12
Married	821	56.23
Partner abroad	2	0.14
Divorced/Separated	25	1.71
Widow/er	37	2.53
Other	33	2.26
**Number of children (N = 1,543)**		
0	479	31.04
1	147	9.53
2	177	11.47
3	147	9.53
4	129	8.36
5	133	8.62
6	99	6.42
7–20	232	15.04
**Status (N = 1,942)**		
Resident	1,589	81.82
Migrant	270	13.90
Other	83	4.27
**Main reason for surgery (N = 1,054)**		
Mine	49	4.65
Bomb explosion	18	1.71
Firearms	684	64.90
Other weapons	134	12.71
Disease	19	1.80
Self-harm	21	1.99
Accident	18	1.71
Other / Not disclosed	111	10.42
**Other factors of vulnerability highlighted by the patient during the first session (not mutually exclusive) (N = 2,008)**		
Destroyed/Lost property and/or income	698	34.76
Mother head of household	54	2.69
Natural death of loved one less than two years ago	182	9.06
Natural death of loved one more than two years ago	317	15.79
Severe or chronic medical/Physical condition	563	28.04
Severe or chronic mental health condition	114	5.68
**Place of violence (N = 1,970)**		
Home	670	31.01
School/Work	327	16.60
On the road/While going somewhere	794	40.30
During combat	82	4.16
Other	97	4.93
**Type of perpetrator (N = 1,855)**		
Known civilian	120	6.47
Unknown civilian	250	13.48
Military/Armed group	1,250	67.39
Other or unknown	235	12.67
**Number of perpetrators (N = 1,159)**		
None	19	1.64
One	456	39.34
Several	684	59.02
**Timing: Days between latest violence and first consultation (N = 1,544)**		
0–2	320	20.73
3–14	843	54.60
15–90	278	18.01
91–365	45	2.91
<365	58	3.76
**Number of individual sessions excluding pre- and post-assessments (N = 1,474)**		
0	4	0.27
1–2	425	28.83
3–4	668	45–32
5–6	285	19.34
7–13	92	6.24
**Length: Days between pre- and post-assessment (N = 1,271)**		
<8 days	316	24.86
8–14 days	350	27.54
15–21 days	223	17.55
22–30 days	153	12.04
>30 days	229	18.02

### Pre- and post-scores of psychological distress and functioning

**Fig 1:**
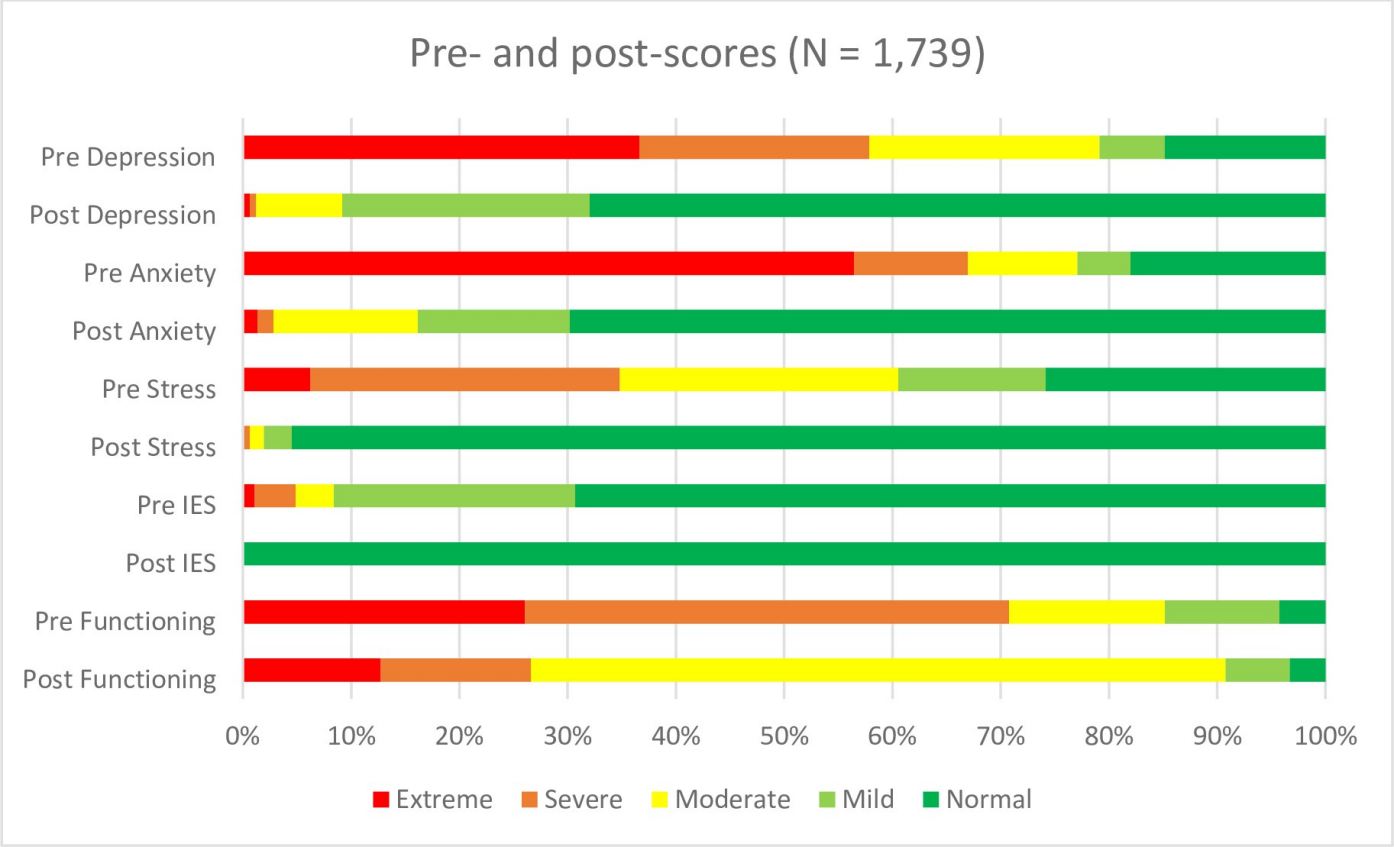
Pre- and post-scores of DASS21, IES-R and ICRC functioning scales.

On the DASS21 used in the DRC and Mali (Table 2), anxiety was the most frequent extreme pre-score among the three sub-scales (56.43%, n = 746), followed by depression (36.61%, n = 484) and stress (5.97%, n = 79). At the time of the post-test, the type of distress that was reduced to normal levels in the greatest number of patients was stress (95.50%, n = 1,082), followed by anxiety (69.81%, n = 791) and depression (67.96%, n = 770). On the IES-R used in Nigeria, only a small proportion showed extreme scores at baseline (1.10%, n = 5) and all patients had normal PTSD scores following MHPSS (100%, n = 314) (see section on sources of data and variables). Lastly, on the ICRC functioning scale used in the DRC and Mali, albeit not systematically, around a quarter of the patients showed extreme difficulties prior to receiving MHPSS (26.06%, n = 172). The number of patients showing extreme difficulties in functioning at the time of post-test was reduced to less than half (12.75%, n = 58) (Fig 1).

**Table 2:** Distress and functioning categories.

Category	Extreme: n (%)	Severe: n (%)	Moderate: n (%)	Mild: n (%)	Normal: n (%)
**DASS21**					
Depression subscale					
Pre-test (N = 1,322)	484 (36.61)	281 (21.26)	281 (21.26)	80 (6.05)	196 (14.83)
Post-test (N = 1,33)	7 (0.62)	7 (0.62)	90 (7.94)	259 (22.86)	770 (67.96)
Anxiety subscale					
Pre-test (N = 1,322)	746 (56.43)	139 (10.51)	134 (10.14)	65 (4.92)	238 (18.00)
Post-test (N = 1,133)	15 (1.32)	17 (1.50)	151 (13.33)	159 (14.03)	791 (69.81)
Stress subscale					
Pre-test (N = 1,323)	79 (5.97)	363 (27.44)	326 (24.64)	173 (13.08)	382 (28.87)
Post-test (N = 1,133)	1 (0.09)	6 (0.53)	15 (1.32)	29 (2.56)	1,082 (95.50)
**IES-R total score**					
Pre-test (N = 453)	5 (1.10)	17 (3.75)	16 (3.53)	101 (22.30)	314 (69.32)
Post-test (N = 314)	0 (0.00)	0 (0.00)	0 (0.00)	0 (0.00)	314 (100.00)
**ICRC functioning scale**					
Pre-test (N = 660)	172 (26.06)	295 (44.70)	95 (14.39)	70 (10.61)	28 (4.24)
Post-test (N = 455)	58 (12.75)	63 (13.85)	292 (64.18)	27 (5.93)	15 (3.30)

Overall, 92.28% of the patients showed a reduction in symptoms on the DASS21, 93.00% showed a reduction in symptoms on the IES-R and 83.04% showed an improvement on the ICRC Africa functioning scale (Table 3).

**Table 3:** Distress and functioning pre- and post-scores.

	Mean (SD)	95% CI	p-value	Range	Median	% who improved
**Pre-DASS (N = 1,376)**	58.36 (29.26)	56.82; 59.91	<0.0001	0–144	66	
**Post-DASS (N = 1,185)**	17.97 (12.45)	17.26; 18.68	<0.0001	0–92	18	
**Difference in DASS (N = 1,166)**	-42.96 (25.53)	-44.43; -41.49	<0.0001	-104–84	-46	92.28
**Pre-IES (N = 353)**	18.79 (11.70)	17.71; 19.87	<0.0001	1–71	17	
**Post-IES (N = 314)**	4.22 (3.08)	3.88; 4.56	<0.0001	0–19	4	
**Difference in IES (N = 300)**	-14.52 (9.79)	-15.64; 13.41	<0.0001	-46–11	-14	93.00
**Pre-Functioning (N = 660)**	4.54 (3.28)	4.29; 4.79	<0.0001	0–14	4	
**Post-Functioning (N = 455)**	7.98 (2.72)	7.72; 8.23	<0.0001	0–14	8	
**Difference in Functioning (N = 454)**	3.51 (2.51)	2.51; 3.74	<0.0001	-5–11	4	83.04

### Determinants of high distress and low functioning prior to MHPSS

Various factors were associated with high distress and low functioning prior to receiving MHPSS (Table 4). On the DASS21 scale, adults were much more likely than children to report high levels of depression at baseline, peaking at 25–34 years of age (aOR 6.44, p = 0.028), and a comorbidity with high anxiety (aOR 5.62, p = <0.0001) and high stress (aOR 6.70, p = <0.0001) were observed. Patients wounded by self-harm appeared much more likely to present high levels of depression at baseline (6.27, p = 0.145). However, this finding was not significant due to the small number of patients falling within this category. Patients wounded by weapons other than firearms were less likely to present high level of depression at baseline (aOR 0.34, p = 0.043).

**Table 4:** Factors associated with high distress and low functioning at baseline.

Variables	cOR (95%CI)	p-value	aOR (95%CI)	p-value
**DEPRESSION (DASS21)**				
**Age**				
0–17	Ref	--	Ref	--
18–24	3.98 (1.66; 9.51)	0.002	4.95 (0.95; 25.88)	0.058
25–34	3.70 (1.55; 8.81)	0.003	6.44 (1.23; 33.77)	0.028
35–44	3.40 (1.42; 8.16)	0.006	6.36 (1.19; 34.12)	0.031
45–85	2.69 (1.11; 6.53)	0.028	5.64 (3.43; 9.27)	0.036
**High anxiety at baseline**				
No	Ref	--	Ref	--
Yes	18.43 (13.91; 24.42)	<0.0001	5.64 (3.43; 9.27)	<0.0001
**High stress at baseline**				
No	Ref	--	Ref	--
Yes	19.33 (14.64; 25.51)	<0.0001	6.70 (4.21; 10.64)	<0.0001
**Main reason for surgery**				
Mine	Ref	--	Ref	--
Bomb explosion	6.54 (0.77; 55.83)	0.086	2.28 (0.16; 31.64)	0.540
Bullet / gunshot	0.98 (0.52; 1.84)	0.938	0.45 (0.19; 1.04)	0.060
Non-automatic weapon	2.26 (1.06; 4.84)	0.036	0.34 (0.12; 0.97)	0.043
Self-harm	1.09 (0.23; 5.17)	0.914	6.27 (0.53; 74.06)	0.145
Accident	2.92 (1.27; 6.69)	0.011	1.58 (0.55; 4.58)	0.396
Other / Not disclosed	0.16 (0.02; 1.59)	0.119	1.28 (0.10; 16.18)	0.847
**ANXIETY (DASS21)**				
**High depression at baseline (N = 1,322)**				
No	Ref	--	Ref	--
Yes	18.43 (13.91; 24.42)	<0.0001	6.43 (4.40; 9.37)	<0.0001
**High stress at baseline (N = 1,322)**				
No	Ref	--	Ref	--
Yes	28.30 (20.91; 38.30)	<0.0001	11.96 (8.18; 17.50)	<0.0001
**Timing: Days between latest violence and first consultation (N = 1,125)**				
0–2	Ref	--	Ref	--
3–14	0.67 (0.49; 0.93)	0.015	0.82 (0.52; 1.32)	0.419
15–90	0.22 (0.15; 0.33)	<0.0001	0.50 (0.28; 0.89)	0.019
91–365	0.12 (0.02; 0.60)	0.010	0.06 (0.00; 0.97)	0.047
>365	0.47 (0.19; 1.16)	0.101	0.58 (0.15; 2.23)	0.431
Linear trend	0.56 (0.48; 0.67)	<0.0001	0.75 (0.59; 0.94)	0.014
**Type of perpetrator (N = 1,228)**				
Known civilian	Ref	--	Ref	--
Unknown civilian	0.46 (0.24; 0.90)	0.023	1.63 (0.61; 4.35)	0.333
Military/Armed group	0.86 (0.46; 1.58)	0.623	2.47 (1.01; 6.05)	0.047
Other or unknown	0.31 (0.15; 0.67)	0.003	1.51 (0.50; 4.52)	0.462
**STRESS (DASS21)**				
**Days between latest violence and first consultation (N = 1,126)**				
0–2	Ref	--	Ref	--
3–14	0.70 (0.52; 0.96)	0.027	0.94 (0.60; 1.47)	0.785
15–90	0.20 (0.14; 0.30)	<0.001	0.44 (0.25; 0.77)	0.004
91–365	0.25 (0.06; 1.04)	0.058	0.81 (0.12; 5.59)	0.835
>365	0.56 (0.23; 1.37)	0.206	0.89 (0.25; 3.11)	0.854
Linear trend	0.57 (0.48; 0.67)	<0.001	0.78 (0.63; 0.98)	0.032
**High depression at baseline (N = 1,322)**				
No	Ref	--	Ref	--
Yes	19.33 (14.64; 25.51)	<0.0001	7.19 (5.01; 10.31)	<0.001
**High anxiety at baseline (N = 1,322)**				
No	Ref	--	Ref	--
Yes	28.30 (20.91; 38.30)	<0.0001	10.74 (7.39; 15.61)	<0.001
**Natural death of a loved one more than two years ago (N = 1,323)**				
No	Ref	--	Ref	--
Yes	7.81 (5.22; 11.68)	<0.001	2.29 (1.37; 3.82)	0.002
**POST-TRAUMATIC STRESS (IES-R)**				
**Education level (N = 451)**				
Illiterate	Ref	--	Ref	--
Basic	2.61 (1.70; 4.08)	<0.0001	1.62 (0.37; 7.10)	0.523
Medium	0.97 (0.49; 1.92)	0.931	0.08 (0.01; 0.91)	0.042
High	2.32 (0.82; 6.59)	0.113	1	
**Main reason for surgery (N = 81)**				
Mine	Ref	--	Ref	--
Bomb explosion	6 (0.35; 101.57)	0.214	3.39 (0.17; 69.19)	0.428
Bullet / gunshot	16.5 (1.09; 205.18)	0.043	21.34 (1.25; 365.64)	0.035
Non-automatic weapon	6 (0.22; 162.53)	0.287	6 (0.22; 162.53)	0.287
Disease	2.4 (0.18; 32.88)	0.512	3.21 (0.21; 48.65)	0.401
Self-harm	10.5 (0.84; 130.66)	0.068	13.03 (0.89; 189.72)	0.060
Accident	0.375 (0.03; 5.57)	0.476	0.32 (0.02; 5.28)	0.425
**FUNCTIONING (ICRC Africa scale)**				
**Education level (N = 658)**				
Illiterate	Ref	--	Ref	--
Basic	0.82 (0.56; 1.22)	0.332	0.93 (0.62; 1.38)	0.708
Medium	0.93 (0.54; 1.61)	0.801	1.07 (0.61; 1.88)	0.805
High	2.37 (0.99; 5.64)	0.052	2.63 (1.07; 6.42)	0.034
**Weapon bearer (N = 660)**				
No or not disclosed	Ref	--	Ref	--
Yes	0.08 (0.01; 0.58)	0.013	0.07 (0.01; 0.57)	0.013

cOR: crude odds ratio

aOR: adjusted odds ratio (adjusted for other variables in the table)

p-value from Wald test

High levels of anxiety correlated with high levels of depression (aOR 6.43, p = <0.0001) and stress (aOR 11.96, p = <0.0001). A linear trend was found between increasing number of days between violence and first consultation and decreased likelihood of presenting high levels of anxiety (aOR 0.75, p = 0.014). Violence committed by military/armed group was associated with increased likelihood of reporting high levels of anxiety (aOR 2.47, p = 0.047).

With regard to symptoms of stress, a similar comorbidity was found with symptoms of depression (aOR 7.19, p = <0.0001) and anxiety (aOR 10.74, p = 0.0001). The timing of the response also played a role as a linear trend was found between increasing number of days between violence and first consultation and decreased likelihood of presenting high levels of stress (aOR 0.78, p = 0.032). Also, the natural death of a loved one more than two years ago increased the likelihood of reporting high levels of stress (aOR 2.29, p = 0.002).

On the IES-R, high scores at baseline were more likely to be found among illiterate patients compared to patients with medium education level (aOR 0.08, p = 0.042). The likelihood of reporting high levels of PTSD was considerably increased among patients having been wounded by firearms (aOR 21.34, p = 0.035) and self-harm (aOR 13.03, p = 0.60).

Finally, with regard to functioning, low scores at baseline were more likely to be found among patients with a high education level (aOR 2.63, p = 0.034) than illiterate patients. Compared to civilians, weapon bearers were much less likely to report low levels of functioning at baseline (aOR 0.07, p = 0.013).

### Determinants of improvement following MHPSS

Several variables correlated with reduced psychological distress and increased functioning following MHPSS (Table 5). On the DASS21, determinants of reduced depression included high depression at baseline (aOR 29.27, p = >0.0001), reduced anxiety (aOR 3.11, p = <0.0001) and reduced stress (aOR 2.49, p = <0.0001). Also, patients having experienced the death of a loved one more than two years ago were more likely to show reduced levels of depression following MHPSS (aOR 2.26, p = <0.0001). The counsellors whose patients were most likely to witness this decrease were characterized by being male and having a high education level.

**Table 5:** Factors associated with improved distress and functioning following MHPSS (cOR: crude odds ratio, aOR: adjusted odds ratio, p-value from Wald test).

Variables	cOR (95%CI)	p-value	aOR (95%CI)	p-value
**DEPRESSION (DASS21)**				
**Improved anxiety (N = 1,079)**				
No	Ref	--	Ref	--
Yes	8.82 (6.70; 11.61)	<0.0001	3.11 (2.00; 4.85)	<0.0001
**Improved stress (N = 1,079)**				
No	Ref	--	Ref	--
Yes	10.66 (8.04; 14.13)	<0.0001	3.72 (2.48; 5.56)	<0.0001
**High depression at baseline (N = 1,079)**				
No	Ref	--	Ref	--
Yes	40.87 (28.36; 58.89)	<0.0001	33.11 (19.89; 55.12)	<0.0001
**Counsellor (N = 987)**				
A	Ref	--	Ref	--
B	0.18 (0.10; 0.30)	<0.0001	0.29 (0.14; 0.59)	0.001
F	3.11 (1.05; 9.23)	0.041	8.44 (2.13; 33.36)	0.002
N	0.04 (0.02; 0.08)	<0.0001	2.73 (1.07; 6.99)	0.035
**Natural death of a loved one more than two years ago (N = 1,079)**				
No	Ref	--	Ref	--
Yes	2.21 (1.59; 3.06)	<0.0001	2.23 (1.12; 4.44)	<0.023
**ANXIETY (DASS21)**				
**Counsellor (N = 989)**				
A	Ref	--	Ref	--
G	0.28 (0.02; 4.55)	0.001	126.86 (6.57; 2450.05)	0.001
K	0.10 (0.05; 0.23)	<0.0001	14.18 (3.41; 58.90)	<0.0001
M	0.09 (0.05; 0.18)	<0.0001	9.82 (2.91; 33.21)	<0.0001
O	0.35 (0.20; 0.62)	<0.0001	0.34 (0.16; 0.73)	0.005
**High anxiety at baseline (N = 1,079)**				
No	Ref	--	Ref	--
Yes	78.63 (49.40; 125.15)	<0.0001	97.65 (42.63; 223.66)	<0.0001
**Improved depression (N = 1,079)**				
No	Ref	--	Ref	--
Yes	10.29 (7.75; 13.66)	<0.0001	3.05 (1.95; 4.77)	<0.0001
**Improved stress (N = 1,079)**				
No	Ref	--	Ref	--
Yes	13.67 (10.18; 18.35)	<0.0001	4.91 (3.10; 7.77)	<0.0001
**STRESS (DASS21)**				
**Age (N = 1,076)**				
0–17	Ref	--	Ref	--
18–24	1.98 (0.69; 5.71)	0.206	0.04 (0.00; 0.26)	0.001
25–34	1.49 (0.52; 4.28)	0.457	0.03 (0.00; 0.23)	0.001
35–44	1.40 (0.48; 4.07)	0.532	0.04 (0.00; 0.27)	0.001
45–85	1.26 (0.43; 3.70)	0.670	0.04 (0.01; 0.30)	0.002
**Counsellor (N = 988)**				
A	Ref	--	Ref	--
B	0.26 (0.15; 0.44)	<0.0001	0.12 (0.05; 0.25)	<0.0001
E	0.31 (0.16; 0.58)	<0.0001	0.33 (0.13; 0.82)	0.017
**High stress at baseline (N = 1,080)**				
No	Ref	--	Ref	--
Yes	42.50 (29.58; 61.08)	<0.0001	25.63 (14.06; 46.74)	<0.0001
**Improved anxiety (N = 1,079)**				
No	Ref	--	Ref	--
Yes	12.10 (9.08; 16.11)	<0.0001	4.39 (2.63; 7.35)	<0.0001
**Improved depression (N = 1,079)**				
No	Ref	--	Ref	--
Yes	10.66 (8.04; 14.13)	<0.0001	3.48 (2.15; 5.62)	<0.0001
**Length of MHPSS: Days between pre- and post-assessment (N = 854)**				
<8 days	Ref	--	Ref	--
8–14 days	1.29 (0.89; 1.88)	1.850	1.24 (0.64; 2.38)	0.526
15–21 days	4.04 (2.59; 6.31)	<0.0001	2.52 (1.11; 5.72)	0.028
22–30 days	5.70 (3.29; 9.89)	<0.0001	2.80 (1.07; 7.32)	0.036
>30 days	4.81 (3.00; 7.71)	<0.0001	2.43 (1.06; 5.61)	0.037
**Post-traumatic stress (IES-R)**				
**Education level (N = 299)**				
Illiterate	Ref	--	Ref	--
Basic	1.93 (1.16; 3.23)	0.012	0.70 (0.23; 2.12)	0.530
Medium	0.73 (0.32; 1.64)	0.444	0.96 (0.17; 5.40)	0.962
High	1.91 (0.60; 6.08)	0.275	8.95 (1.26; 63.78)	0.029
**High IES-R scores at baseline (N = 300)**				
No	Ref	--	Ref	--
Yes	180	<0.0001	202.23 (68.07; 600.77)	<0.0001
**Length of MHPSS: Days between pre- and post-assessment (N = 277)**		--		
<8 days	Ref	0.001	Ref	--
8–14 days	3.86 (1.76; 8.45)	<0.0001	.74 (0.41; 7.34)	0.451
15–21 days	8.01 (3.41; 18.81)	<0.0001	4.60 (0.91; 23.36)	0.065
22–30 days	11.05 (4.57; 26.73)	<0.0001	8.73 (1.74; 43.80)	0.008
>30 days	6.84 (3.14; 14.93)		2.86 (0.69; 11.89)	0.147
**FUNCTIONING (ICRC Africa scale)**				
**Age (N = 452)**				
0–17	Ref	--	Ref	--
18–24	1.90 (0.92; 3.91)	0.081	1.94 (0.79; 4.)	0.147
25–34	1.65 (0.83; 3.24)	0.150	1.83 (0.79; 4.27)	0.160
35–44	2.09 (1.02; 4.28)	0.043	3.74 (1.53; 9.16)	0.004
45–85	1.33 (0.66; 2.69)	0.425	1.46 (0.61; 3.50)	0.397
**Low functioning at baseline (N = 454)**				
No	Ref	--	Ref	--
Yes	14.12 (8.97; 22.22)	<0.0001	15.82 (9.80; 25.56)	<0.001
**Severe or chronic medical/physical condition**				
No	Ref	--	Ref	--
Yes	1.82 (1.22; 2.71)	0.003	1.66 (1.01; 2.73)	0.044

cOR: crude odds ratio

aOR: adjusted odds ratio (adjusted for other variables in the table)

p-value from Wald test

Reduced levels of anxiety correlated with high anxiety at baseline (aOR 97.14, p = <0.0001) as well as reduced depression (aOR 2.98, p = <0.0001) and stress (aOR 4.93, p = 0.0001) following MHPSS. An association was also observed between reduced anxiety and counsellors characterized by a high educational level, male gender and/or 40 years or older. Factors negatively associated with improved anxiety included lack of social support (aOR 0.17, p = 0.047) and suffering from a severe or chronic medical condition (aOR 0.40, p = 0.013).

Reduced stress levels following MHPSS were associated with having reported high levels of stress at baseline (aOR 25.69, p = <0.0001) and reduced anxiety (aOR 4.39, p = <0.0001) and depression (aOR 3.48, p = <0.0001) following MHPSS. Minors were much more likely than all adult age groups to show reduced stress after the MHPSS. Also, a linear trend was found between the length of the response and the likelihood of reduced stress. Certain counsellors correlated with a decreased likelihood of reduced stress levels. These counsellors were characterized by being female and below 40 years of age.

On the IES-R, the strongest predictor of improvement was having high PTSD scores at baseline (aOR 202.23, p = <0.0001), i.e. having greater room for improvement. Also, patients with reduced IES-R scores were more likely to have a higher education level (aOR 8.95, p = 0.029) and to have received MHPSS that lasted between 22 and 30 days (aOR 8.73, p = 0.008).

Finally, when it came to improved daily functioning, predictors included having reported low levels of functioning at baseline (aOR 15.82, p = <0.001) as well as suffering from a severe or chronic medical/physical condition (aOR 1.66, p = 0.044). The age group most likely to improve its functioning was 35–44 years (aOR 3.74, p = 0.004).

## Discussion

The findings of this study were particularly rich and diverse. Levels of psychological distress and functioning prior to and following MHPSS were associated with numerous characteristics of the patient and of the response itself.

### Characteristics of the patient

#### Bereavement

Having experienced the natural death of a loved one stood out as a distinct vulnerability factor that increased the likelihood of reporting high levels of stress prior to MHPSS.

#### Education level

Patients with a higher educational level were much more likely to report reduced PTSD symptoms following MHPSS. The link between high education level and well-being as mediated by psychosocial resources has been documented in other settings [9] but would merit further investigation among war-wounded patients. Efforts should be made to better tailor the MHPSS to war-wounded patients with lower levels of education.

#### Gunshot

Injury from gunshots correlated with increased PTSD symptoms prior to MHPSS. This points to the pertinence of a systematic referral of this sub-group of patients to psychological support. The fact that injury from gunshots did not correlate negatively with treatment outcome indicates that the MHPSS in its current form adequately addresses the needs of patient having suffered this type of injury.

#### Minors

Prior to MHPSS, war-wounded patients aged 0–17 were less likely to report high levels of depression. Following MHPSS, minors were more likely to show reduced stress and more likely to show increased functioning. As the monitoring tools used were designed for adults, it is uncertain whether they were as appropriate for minors as, for example, the Paediatric Emotional Distress Scale (PEDS) [10] would have been.

#### Perpetrator

Violence committed by the military or armed groups was a predictor of increased anxiety prior to MHPSS. This is consistent with the findings of a study among victims of violence in health facilities in similar settings [8] and points to the need to pay attention to the perpetrator profile as a potential vulnerability factor.

#### Severe or chronic medical condition

Patients suffering from a severe or chronic medical condition were less likely to show reduced anxiety and increased functioning following MHPSS. While the severity of the medical condition would explain the fact that functioning does not significantly improve during the stay in the hospital, there could nonetheless be a need to more thoroughly address during counselling the anxiety experienced by this sub-group, whether health-related [11] or otherwise, and to offer long-term follow-up.

#### Self-harm

High levels of depression and PTSD prior to MHPSS was associated with wounds caused by self-harm. This finding is likely to be a case of reversed causality whereby depression and PTSD may lead to self-harm. Similarly, in Northern Ireland, more than 50% of individuals with PTSD reported deliberate self-harm [12]. Among veterans in the United States, a study found evidence of an interaction between PTSD and depression diagnoses in predicting intentional self-harm [13]. Particular attention will need to be given to this minority of patients, focusing on the circumstances, thoughts and feelings that led to the self-harm as well as the management of thoughts of self-harm and/or suicide in the future.

#### Social support

Patients lacking social support were less likely to show reduced anxiety following MHPSS. This is consistent with other studies pointing to social support as a protective factor with regard to mental health in general [14] and anxiety disorder in particular [15]. It would, therefore, seem pertinent to include caregivers (even) more in the MHPSS offered to war-wounded patients with the aim of increasing both actual and perceived social support.

### Characteristics of the intervention

#### Counsellor profile

Certain counsellors unexpectedly stood out as correlating with increased or decreased likelihood of improvement among their patients. Counsellors associated with patients who showed considerable improvement following MHPSS where characterized by being male, having a high educational level (university studies) and being 40 years or older. Likewise, counsellors whose patients were less likely than average to improve tended to be young and female, although not necessarily less educated. Determining to what extent these findings can be explained by the fact that most of the patients were male and/or prefer a certain type of counsellor would require further investigation, possibly through a qualitative study of the styles and techniques of each category of counsellors.

#### MHPSS timing

The earlier the support is offered, the greater the likelihood of the patient reporting acute distress. Given that psychological needs are at their highest in the days following war injury, efforts should be made to initiate MHPSS to patients at the time when they are most vulnerable.

#### MHPSS length

From a duration of three weeks onwards, the likelihood of positive MHPSS outcomes increased considerably. This indicates that meaningful response could be offered during a relatively short period of time.

#### Monitoring distress

When measuring the psychological distress of war-wounded patients in Africa, the use of the DASS21 appears preferable to the IES-R. With more than two-thirds of the patients obtaining normal pre-scores on the IES-R and all patients obtaining normal post-scores, the DASS21 stands out as the most sensitive tool in terms of measuring variation and differentiating between patients with regard to their level of psychological distress.

### Strengths and limitations

As the first-of-its kind looking at ICRC MHPSS for more than 2,000 war-wounded patients in conflict settings in Africa, the real-life settings, the uniqueness of the data and the large number of patients involved constitute major strengths of this study. Furthermore, the quality of the data derived from standardized psychometric tools and following each individual patient before and after MHPSS may be considered as important attributes.

The main limitation of this study is the absence of a control group. As in any observational study, it cannot be stated with certainty whether the observed correlations involve an element of *causation*, i.e. whether it was the MHPSS that improved the patients’ well-being or it was simply the passage of time or other factors that led to the observed outcomes. Another limitation is the fact that all the data used in the study stems from information obtained from the patient him or herself. A bias may have been present due to the absence of objective medical information regarding, for example, the gravity of the patients’ wounds.

The results of this study highlight the pertinence of addressing MHPSS needs alongside the medical needs arising from severe injury in the context of armed conflict. Just as psychological distress may hamper post-surgical recovery, so a healthy mental health status promotes physical healing. Certain characteristics of the patient and the MHPSS itself call for further discussion to informed clinical practice.

### Summary of recommendations

#### Triage/prioritization

- Offer extended follow-up to patients with a severe or chronic medical condition and/or wounds caused by self-harm

#### Clinical implications

Involve caregivers (even) more in the MHPSS to increase social support

To improve monitoring and inform decision-making:

- Use the DASS21 rather than the IES-R to monitor distress, as it appears to differentiate better between patients
- Use age-appropriate tools to monitor distress, such as the PEDS
- Monitor the therapeutic techniques used during counselling
- Monitor characteristics of the counsellor

#### Further research

- Compare MHPSS outcomes with medical outcomes
- Conduct a qualitative study of the individual styles and techniques used by the counsellors
- Conduct a qualitative study to explore the role of the patient’s education level in benefiting from MHPSS

## Supporting information

S1 Appendix (DOCX)
