# Supplementary material for: Mental health and psychosocial support for the war-wounded: A retrospective cohort study from the Democratic Republic of Congo, Mali and Nigeria
Source: PLoS One. 2022 May 24;17(5):e0268737. doi: 10.1371/journal.pone.0268737 (PMC9128955; doi:10.1371/journal.pone.0268737)
Supplement: S1 Appendix — (DOCX) [file pone.0268737.s001.docx]

**Appendix**

**Appendix I:** Distress and Functioning categories

| **Category** | **Extreme:**  **n (%)** | **Severe:**  **n (%)** | **Moderate:**  **n (%)** | **Mild:**  **n (%)** | **Normal:**  **n (%)** |
| --- | --- | --- | --- | --- | --- |
| **DASS21**  Depression subscale  Pre-test (N=1,322)  Post-test (N=1,33)  Anxiety subscale  Pre-test (N=1,322)  Post-test (N=1,133)  Stress subscale  Pre-test (N=1,323)  Post-test (N=1,133) | 484 (36.61)  7 (0.62)  746 (56.43)  15 (1.32)  79 (5.97)  1 (0.09) | 281 (21.26)  7 (0.62)  139 (10.51)  17 (1.50)  363 (27.44)  6 (0.53) | 281 (21.26)  90 (7.94)  134 (10.14)  151 (13.33)  326 (24.64)  15 (1.32) | 80 (6.05)  259 (22.86)  65 (4.92)  159 (14.03)  173 (13.08)  29 (2.56) | 196 (14.83)  770 (67.96)  238 (18.00)  791 (69.81)  382 (28.87)  1,082 (95.50) |
| **IES-R total score**  Pre-test (N=453)  Post-test (N=314) | 5 (1.10)  0 (0.00) | 17 (3.75)  0 (0.00) | 16 (3.53)  0 (0.00) | 101 (22.30)  0 (0.00) | 314 (69.32)  314 (100.00) |
| **ICRC functioning scale**  Pre-test (N=660)  Post-test (N=455) | 172 (26.06)  58 (12.75) | 295 (44.70)  63 (13.85) | 95 (14.39)  292 (64.18) | 70 (10.61)  27 (5.93) | 28 (4.24)  15 (3.30) |
